# Detecting abnormal cell behaviors from dry mass time series

**DOI:** 10.1038/s41598-024-57684-w

**Published:** 2024-03-25

**Authors:** Romain Bailly, Marielle Malfante, Cédric Allier, Chiara Paviolo, Lamya Ghenim, Kiran Padmanabhan, Sabine Bardin, Jérôme Mars

**Affiliations:** 1 Univ. Grenoble Alpes, CEA, List, F-38000 Grenoble, France; 2 Univ. Grenoble Alpes, CEA, Leti, F-38000 Grenoble, France; 3 Janelia Research Campus, Howard Hughes Medical Institute, Ashburn, VA USA; 4 Univ. Grenoble Alpes, INSERM, CEA-IRIG, BGE, Biomics, F-38000 Grenoble, France; 5 Institut de Génomique Fonctionnelle de Lyon, Univ. Lyon, CNRS/ENS, UMR 5242, Lyon, France; 6 Institut Curie, PSL Research University, CNRS, UMR 144, Molecular Mechanisms of Intracellular Transport, F-75005 Paris, France; 7 Univ. Grenoble Alpes, CNRS, Grenoble-INP, GIPSA-Lab, 38000 Grenoble, France

**Keywords:** Machine learning, Cell biology, Machine learning, Cell biology

## Abstract

The prediction of pathological changes on single cell behaviour is a challenging task for deep learning models. Indeed, in self-supervised learning methods, no prior labels are used for the training and all of the information for event predictions are extracted from the data themselves. We present here a novel self-supervised learning model for the detection of anomalies in a given cell population, StArDusTS. Cells are monitored over time, and analysed to extract time-series of dry mass values. We assessed its performances on different cell lines, showing a precision of 96% in the automatic detection of anomalies. Additionally, anomaly detection was also associated with cell measurement errors inherent to the acquisition or analysis pipelines, leading to an improvement of the upstream methods for feature extraction. Our results pave the way to novel architectures for the continuous monitoring of cell cultures in applied research or bioproduction applications, and for the prediction of pathological cellular changes.

## Introduction

Predicting the future behavior of single cells within a large population is an interesting task for deep learning models. To this end, different approaches have already been developed to predict cell trajectories from transcriptomics dataset^1^, detect cells that deviate from normal behavior in time-lapse imaging^2^, and learning cell competition rules^3^. Once a complete behavioral pattern is known within a temporal interval, any deviation from “normality” can be classified as “abnormal”. This is of crucial importance when monitoring a cell population, as identifying cells that harbor cancerous mutations based on deviation from a “typical” trajectory would allow for predictions about pathological changes.

Recently, novel techniques in bioimaging coupled to advanced deep learning models have allowed the visualization, quantification, and monitoring of important cellular features over time on large populations and in label-free conditions^4^. Lensfree phase microscopy allows indeed the extraction of important cellular features over time, such as the dry mass (i.e. the weight of the cell’s content other than water). This parameter is a critical biological feature, as it remains invariant over different cell generations and reflects the growth and division stages of a cell. Dry mass is therefore key to the understanding of cellular behavior, including cell size, cycle, state, and homeostasis^5–7^. Monitoring the dry mass of a cell over time is thus a proxy of the overall cellular status, and could allow the prediction about possible abnormal deviations.

We propose a method using self-supervised learning to detect abnormalities or anomalous cell behavior on temporal trajectories of dry mass. Self-supervised learning is a recent training paradigm that does not need any label to train a machine learning model. It uses information extracted from the data themselves, called pseudo-labels. These pseudo-labels are used to train a neural network to perform a pretext task, that can then be used in the downstream pipeline, in our case anomaly detection. Forecasting the future dry mass of a cell is the pretext task used in this paper.

Unsupervised (i.e. no labels are manually provided to the model) anomaly detection has been used lately in a wide variety of domains^8^, including but not limited to, astronomy^9^, earth science^10^, neuroscience^11^, oceanography^12^ or physics^13,14^. We focused on anomaly detection on time series without any prior labels as presented by Gupta et al.^15^. Here, the detection is achieved with a two stage anomaly detector, relying on the comparison of the measured and predicted nominal trajectory.

We call the proposed model StArDusTS, for Self-supervised Anomaly Detection on Time Series, and use it on distinct datasets of cellular dry mass time series. To assess its performances, we designed an experimental validation using different cell lines, *i.e.* a human cancer HeLa cell line and murine fibroblasts cells, cultured and imaged in different laboratories. Overall, we report a precision of 96% in the automatic detection of anomalies present in these different datasets. Several types of biological anomalies from the measurement of cell dry mass alone and without any human priors were detected in the different time-lapses, *e.g.* cells dividing to three cells, very large cells and cell fusion. Additionally, anomaly detection was associated not only with abnormal cell behavior but also with cell measurement errors inherent to the acquisition or analysis pipelines, such as segmentation and tracking. This could lead to an improvement of the upstream methods for cell imaging and analysis. Our results pave the way to novel architectures for the continuous monitoring of cell cultures in applied research or bioproduction applications.

## Method

Here we describe the cell imaging and analysis pipeline in section 2.1, the architecture of the StaArDusTS model in section 2.2 and the different experiments conducted to validate the algorithms. Importantly, we designed an experimental plan featuring three sets of live cell acquisitions conducted in different laboratories.

### Lensfree microscopy for cell dry mass measurement

Lens-free microscopy is a technique providing large field of view, i.e. several $\text {mm}^2$. It has been first developped by Ozcan et al.^16^. Later it has been applied to the live-imaging of cell culture^17^ and their analysis through machine learning methods^4^. Using this method, thousands of cells are simultaneously imaged over several days. The obtained dataset allows the tracking of thousands of individual cells over several tens of hours. Furthermore, it allows the computation of the cell dry mass through the measurement of the optical path difference (OPD) introduced by the sample^17–19^. OPD is measured by the integral of the sample’s refractive index along the optical path.

1 $$\begin{aligned}{} & {} \varphi (x,y)_{\text {shift}}=(\varphi (x,y))-(\varphi _{\text {medium}}) \end{aligned}$$

2 $$\begin{aligned}{} & {} {{\,\mathrm{\text {OPD}}\,}}(x,y) = \lambda \frac{\varphi _{\text {shift}}(x,y)}{2 {\pi }} = \int _{0}^h [n(x,y,z)-n_{\text {medium}}] \,dz \end{aligned}$$

where *n* is the local sample refractive index and $n_{\text {medium}}$ the surrounding medium refractive index, *z* the position along the optical axis, *h* the thickness of the sample and ${\lambda }$ the illumination wavelength^18^. The optical volume difference (${{\,\mathrm{\text {OVD}}\,}}$) is obtained by integrating the ${{\,\mathrm{\text {OPD}}\,}}$ over the total projected area. The OVD can then be converted into cell dry mass according to Eq. (3). In our notation, it is a function of ${\alpha }$, the specific refractive increment which relates the refractive index change to the increase in mass density^20^. The specific refractive index of the different intracellular substances falls with a narrow range, allowing the definition of a constant ${\alpha }$ of 0.18 ${\mu }m^{3}\cdot pg^{-1}$ for most eukaryotic cells^20^.

3 $$\begin{aligned} {{\,\mathrm{\text {OVD}}\,}}= & {} \int _{S} {{\,\mathrm{\text {OPD}}\,}}(x,y) \,dx \,dy \end{aligned}$$

4 $$\begin{aligned} {{\,\mathrm{\text {CDM}}\,}}= & {} \frac{{{\,\mathrm{\text {OVD}}\,}}}{\alpha } \end{aligned}$$

**Figure 1:**
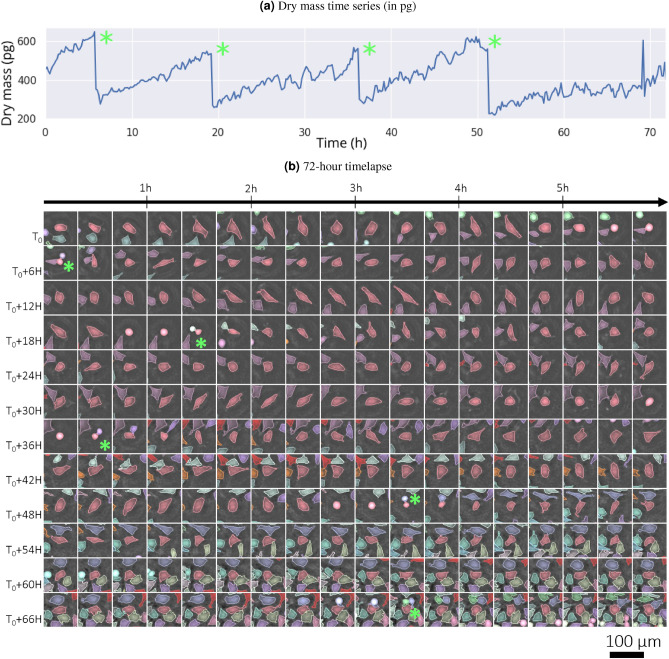
(**a**) Dry mass measurements (in pg) for the cell of interest as a function of time. (**b**) 72-h timelapse acquisition of a HeLa cell. Cell tracking and cell segmentation are computed together to obtain this time-lapse series. Each cell successfully tracked is depicted with a different color. Every cropped image is $100\times 100\mu$ m$^2$. The cell of interest is centered in the cropped image (light red). Four green asterisks point cell divisions. For readability purposes, the time between two images is 20 min, even if the acquisition rate is one image every 10 min. The four cell divisions are associated with a decrease in cell dry mass by a factor of two.

The dry mass measurements have been obtained using a previously described cell imaging analysis pipeline^4^. The latter includes the acquisitions of raw images with a lensfree microscope at a frame rate of one acquisition every 10 min, the reconstruction of ${{\,\mathrm{\text {OPD}}\,}}$ images with the algorithm described in^21^, the detection of the cell with a dedicated 2D-CNN and tracking of each individual cell by means of Fiji plugin Trackmate^22^ and a cell segmentation performed by a watershed-algorithm. For the cell dry mass measurement, several sources of noise are present, *i.e.* in the acquisition, the reconstruction of the ${{\,\mathrm{\text {OPD}}\,}}$, the cell detection, and segmentation. The measured cell dry mass values are in the order of a few hundreds of picograms (pg), while the precision of our measurements was estimated to be about 35 pg^19^. Figure 1 shows an example of the cell imaging analysis pipeline in terms of segmentation (a) and cell dry mass time series (b). Figure A in supplementary materials shows an example of lensfree microscopy acquisition before the segmentation and tracking of cells.

### StArDusTS model

To eliminate any potential human bias when identifying abnormal cells, we introduce the StArDusTS model, which leverages artificial intelligence to autonomously acquire insights from cellular data and identify abnormal patterns within it. StArDusTS, an acronym denoting “Self-supervised Anomaly Detection on Time Series”, comprises two independent algorithmic components. The first component extracts a representation (*i.e.* feature vector, embeddings) from the raw time series to facilitate the detection of abnormal cells. This learned representation is subsequently channeled into the model’s second component, the anomaly detection module, detailed after.

#### Representation learning block

**Self-supervised learning:** Traditionally, supervised learning^23,24^ has been the dominant paradigm in machine learning, where models are trained on meticulously labeled datasets. However, this labeling process is often labor-intensive, time-consuming, expensive, and can induce human biases. Self-supervised learning, on the other hand, seeks to alleviate these limitations by enabling models to learn directly from the data itself.

At its core, self-supervised learning operates on the principle of leveraging inherent structures and relationships within the data^25,26^. It does so by creating surrogate tasks, also called pretext tasks, that generate pseudo-labels or objectives from the input data. These pretext tasks require the model to predict missing parts of the data, reorder sequences, or otherwise make sense of the information it encounters. By solving these tasks, the model learns to capture meaningful features and representations that are useful for downstream tasks, such as image classification, language understanding, recommendation systems, or here, anomaly detection.

Examples of pretext tasks in computer vision include the reconstruction of an image^27,28^, namely auto-encoders, the prediction of rotation of an image^29^, the coloring of black and white images^30^, image inpainting^31^ or even artifact detection^32^.

**Pretext task adapted to time series :** In the context of StArDusTS, we propose the utilization of time series prediction as a pretext task. Specifically, we partition 30-h (180-points) windows of cell dry mass data into two segments: an input segment and a corresponding label. The initial 20 h (120 points) of data serve as the input for the proposed neural network. It is tasked with forecasting the subsequent 10 h (60 points) of cell dry mass, knowing those first 20 h.

The length of the window has been chosen in order to always include a cell division inside the input window. If a smaller input window had been chosen, the cell division would have been a rare event in an input and would therefore not have been learned well, since it would have been subject to catastrophic forgetting^33^. It should also be noted that the decision to exclude mother-daughter cell acquisitions lasting less than 30 h may introduce a potential bias into our algorithm. This bias arises from the restriction to studying only cells with sufficiently extended lifespans. Nevertheless, this selection was imperative to enable the model to acquire a meaningful representation of the dataset.

**Evaluation metric for prediction:** To evaluate the predictive performance, we use the Mean Squared Error ($\mathcal {M}\mathcal {S}\mathcal {E}$) metric, as expressed in Eq. (5). In this equation, *y* is the actual value of the time series, $\widehat{y}$ denotes the predicted values, $y_t$ and $\widehat{y_t}$ are the actual and predicted values at each time step *t*, and $N=60$ corresponds to the number of time steps in a prediction. A lower value of $\mathcal {M}\mathcal {S}\mathcal {E}$ indicates superior predictive performances.

5 $$\begin{aligned} \mathcal {M}\mathcal {S}\mathcal {E}(y, \widehat{y}) = \frac{1}{N} \sum _{t=1}^{N} \left( y_t - \widehat{y_t} \right) ^ 2 \end{aligned}$$

**Neural network architecture:** In conjunction with our choice of a pretext task, it is imperative that we meticulously define the architecture of the AI model that will be assigned with this task. The architecture dictates the network’s structure, layer configurations, and parameter settings, all of which play a pivotal role in the model’s ability to extract meaningful patterns and make accurate predictions.

A one-dimensional convolutional neural network (1D-CNN) is a deep learning architecture designed specifically for processing one-dimensional data, such as time series. Unlike traditional convolutional neural networks (2D-CNN) that operate on two-dimensional grids, 1D-CNNs convolve filters over a single axis, typically time or sequence steps. In a 1D-CNN, convolutional layers are responsible for sliding a small set of learnable filters over the input data, capturing local patterns and features. These filters can detect characteristics like edges, gradients, or more complex temporal patterns in the data. Subsequent layers, such as pooling and fully connected layers, help consolidate these features and enable the network to learn high-level representations^34^.

The choice of the 1D-CNN was motivated by its use in a wide variety of time series applications such as ECG classification^35,36^, fault detection^37–42^, or speech recognition^43,44^. 1D-CNN for time series processing is beneficial because it can capture local patterns and dependencies in the data through its convolutional filters, making it effective for tasks like feature extraction and anomaly detection.

**Figure 2:**
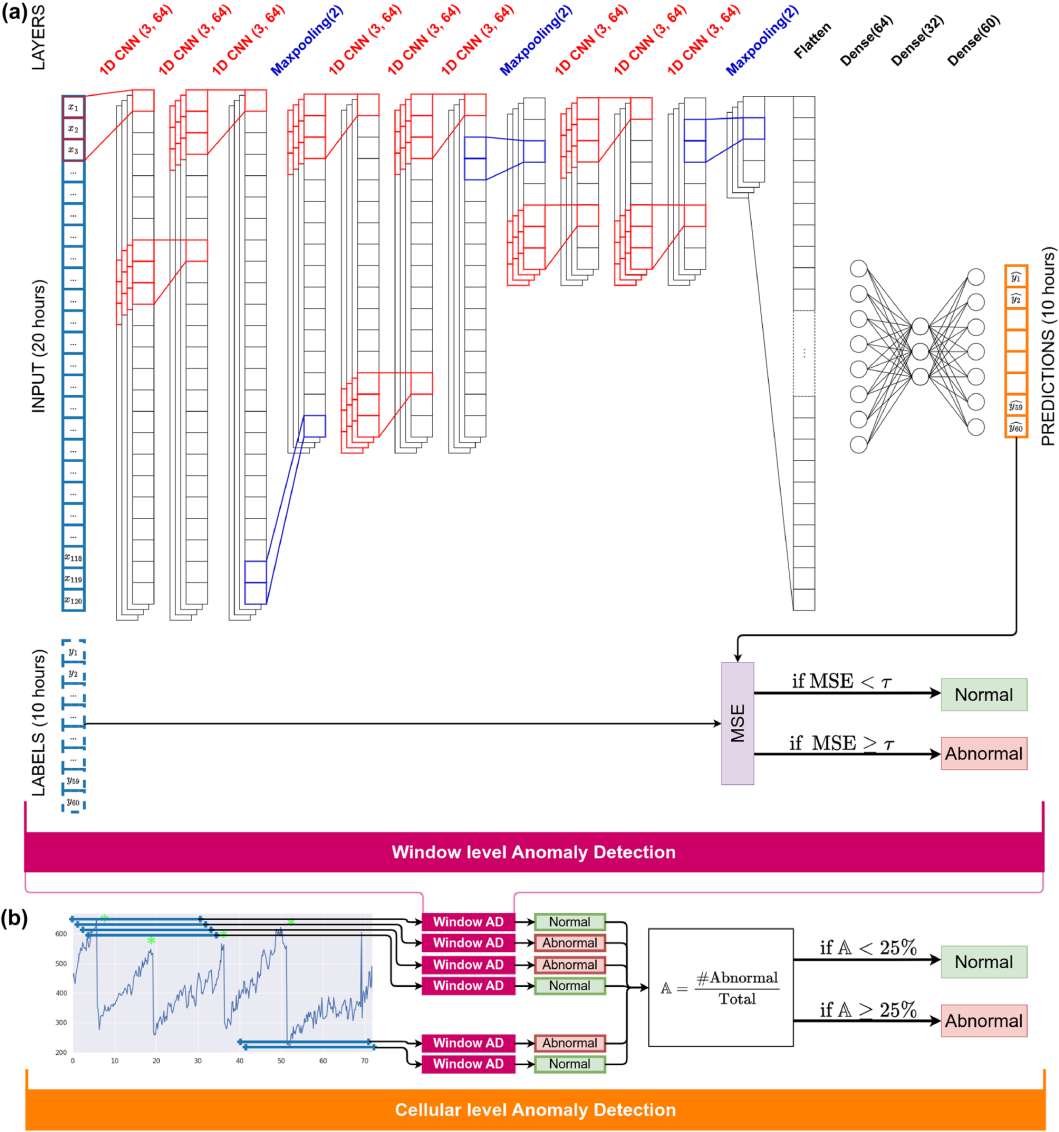
Full scheme of the StArDusTS model. (**a**) Shows the window-wise anomaly detection based on the representation extracted with a 1D-CNN. (**b**) Shows the whole StArDusTS model for a cell, including the 1D-CNN representation learning block, the window-wise anomaly detection and the aggregation of window-wise results with the anomaly score.

The 1D-CNN architecture used in this study is presented Fig. 2a) and contains 3 blocks of 3 Conv1D layers with 64 kernels of size 3, paired with tanh activation functions. The blocks of 3 1D-CNN are separated with maxpooling layers of size 2. The features are then fed in dense layers of size 64 and 32 with reLu activation functions and finally an output layer of size 60. This architecture was selected after a manual optimization of hyperparameters of 1D-CNN^45^.

#### Anomaly detection block

The representation learned by the 1D-CNN is then fed to the anomaly detection block. For this application of detection of abnormal cells, we propose two complementary detectors, the second one working on top of the first one.

**Window level anomaly detection:** A first threshold detector is used to detect the anomalies within a single predicted window. It is based on the value of the prediction metric. The $\mathcal {M}\mathcal {S}\mathcal {E}$ computes the $l_2$ distance between the prediction and the actual value of the future cell dry mass. The larger the $\mathcal {M}\mathcal {S}\mathcal {E}$, the larger the error of the predictor. Selecting the windows with the larger values of metric is therefore selecting the most anomalous windows.

The threshold $\tau _w$ determining which prediction windows have to be considered anomalous is computed such that windows with metric value outside the 95% confidence interval are anomalous. The threshold $\tau _w$ is computed Eq. (6) with $\mu _{\text {training}}$ and $\sigma _{\text {training}}$ respectively the mean and standard deviation of the $\mathcal {M}\mathcal {S}\mathcal {E}$ of the training set.

6 $$\begin{aligned} \tau _w = \mu _{\text {training}} + 2\cdot \sigma _{\text {training}} \end{aligned}$$

**Cellular level anomaly detection:** The detection of abnormal cells consists in the aggregation of multiple window-wise anomalies. For instance, the dry masses of the mother cell and its daughter cells are observed for 50 h. This full length time series is seen independently through 121 overlapping windows of 30 h by the threshold detector. In order to aggregate all the window level detections into a full cellular level, we build a second detector on top of the first one.

The anomaly score $\mathbb {A}$, Eq. (7), uses all the results of detection of all the windows extracted of a cell dry mass and computes a single score from them. This score is the ratio of abnormal windows to the total number of windows of this full-lengthed time series. Cells with a higher score are expected to be *more abnormal* than those with a lower score.

7 $$\begin{aligned} \mathbb {A} = \frac{\# \text { of abnormal windows}}{\text {total } \#\text { of windows}} \end{aligned}$$

Figure 2a shows the window-wise anomaly detection based on the representation extracted with a 1D-CNN and Fig. 2b shows the StArDusTS model for a whole time series, including the 1D-CNN representation learning block, the window-wise anomaly detection with the threshold detector and the aggregation of window-wise results with the anomaly score.

### Experimental plan and constructed datasets

#### Cell culture acquisition

We designed a set of three live cell time-lapses to validate the models. To obtain a first assessment, a set of acquisitions *a* were performed with HeLa cells cultured and imaged in the laboratory. To test generalization, the model trained based on acquisitions *a* have been applied to another set of acquisitions *b*. The latter was obtained with HeLa cells cultured and imaged in a second laboratory. This is a strong generalization test since there are differences between the HeLa cell lines but also between the cell culture protocols used in the two different laboratories.

Finally, with a last set of acquisitions *c*, we could train a deep learning model with a wild type murine fibroblast cell line and test it on abnormal mutated fibroblasts *d*. Acquisitions *c* and *d* were conducted in the same laboratory.

- HeLa cell culture (dataset *a*) comming from the ATCC catalog. HeLa cells were cultured in high glucose Dulbecco’s Modified Eagle Medium (DMEM) supplemented with GlutaMAX, pyruvate, and 10% (v/v) calf serum (Gibco). Cells were grown onto 35 mm glass bottom (0.17 mm) dishes and imaged every 10 min on a Cytonote 1W (Iprasense) for 24–48 h at 37 $^{\circ }\hbox {C}$ and 5% $CO_2$ .
- HeLa cell culture (dataset *b*) donated by Dr Dimitrios Skoufias of the *Institut de biologie structurale* in Grenoble. These cells were grown in DMEM supplemented with GlutaMAX,10% (v/v) heat-inactivated fetal calf serum (FCS), and 1% penicillin and streptomycin. For imaging, 6-well glass bottom culture plates were coated with fibronectin (25 $\upmu \hbox {g}/\hbox {mL}$) for 1 h. Cells were seeded at a concentration of $2 \cdot 10^4$ cells per well and imaged on a Cytonote 6W (Iprasense) every 10 min at 37 $^{\circ }\hbox {C}$ and 5% $CO_2$
- For acquisitions *c* and *d*, wild type mouse fibroblasts were isolated from C57BL/6 mice (acquisition *c*) while Per0 fibroblasts were isolated from $\textit{Period1 (mPer1}^{ldc\text {-}/\text {-})}$; $\textit{Period2 (Per2}^ {ldc\text {-}/\text {-})}$; $\textit{Period3 (mPer3}^{\text {-}/\text {-})}$ triple knock-out mice (PMID:35606517, (acquisition *d*))^46^. All cell lines were cultured in standard DMEM (high glucose) supplemented with 10% FCS (Thermo Fisher), penicillin (25 units/mL, Thermo Fisher) and streptomycin (25 units/mL, Thermo Fisher). Cells were passaged following trypsinization at low density ($2-5 \cdot 10^4$) onto 35mm glass bottom (0.17 mm) dishes (FD-35, Fluoro-dish WPI) and imaged every 10 min for 24–96 h after attachment on a Cytonote 1W (Iprasense) housed inside standard cell culture incubator at a controlled temperature and humidity.

#### Construction of the datasets

The dry mass time series obtained on the basis of the different acquisitions mentioned above have been post-processed to train and validate the models. The time series are split into training, validation and test datasets containing respectively 80%, 10% and 10% of the data points^24^. These *sub*-dataset have been independently normalized such that their mean value is 0 and their standard deviation is 1. Mother-daughter tracks shorter than 30 h are discarded. We used a sliding window, 30 h wide, moved along the full length time series to generate numerous slightly modified versions of the same time series at a 10-min interval difference. This augmentation technique^47^ is used to maximize the data fed to neural networks in order to better learn the phenomenon. Finally, each 30-h window is split in two segments: the first 20 h that will be given as inputs to the neural network and the last 10 h to be predicted and therefore unknown to the neural network. The algorithms must predict the value of the dry mass during this next 10 h. A 1-h sliding window is used to smooth the 10-h signals to be predicted. A summary of the respective number of time series in the splits of each dataset is available in Table 1.

**Table 1:** Train/Validation/test distribution for all four datasets.

Acq	Dataset	Cell line	Location	Number of	Total	Train (80%)	Validation (10%)	Test (10%)
*a*	$\mathscr {A}$	HeLa	Grenoble CEA	Cell trajectories	36,396	29,118	3639	3639
				Cells trajectories $> 30$ h	6700	5520	621	559
				30 h time series	559,141	440,930	60,179	58,032
*b*	$\mathscr {B}$	HeLa	Paris Curie institute	Cell trajectories	27,528	22,024	2752	2752
				Cells trajectories $> 30$ h	7283	5859	706	718
				30 h time series	834,918	665,280	86,363	83,275
*c*	$\mathscr {C}$	Fibroblasts Wild Type	Lyon École Normale Supérieure	Cell trajectories	28,243	22,596	2823	2824
				Cells trajectories $> 30$ h	3986	3169	404	413
				30 h time series	156,381	123,950	16,259	16,172
*d*	$\mathscr {D}$	Fibroblasts Knock Out	Lyon École Normale Supérieure	Cell trajectories	5035			5035
				Cells trajectories $> 30$ h	315			315
				30 h time series	26,688			26,688

For each one, the total number of cells, the numbers of cells with a life-span over 30 h and the number of extracted cell dry mass 30 h time series are reported.

## Results and discussion

### Experiment 1 : Anomaly detection

The purpose of this first experiment is to assess the anomaly detection performances of the StarDusTS model. Therefore, the algorithm is trained, validated and tested on the same datasets. The aim is to check whether the StArDusTS model is capable to learn a representation and detect anomalies among the same cell culture. This experiment is run on both dataset $\mathscr {A}$ and $\mathscr {B}$. In order to assess the performances of the detection, some of the videos were manually analyzed to detected abnormal cells. These labels allowed the assessement of the precision (i.e. the ratio between the anomalies and the detections) of the model.

After training, the StArDusTS models raised respectively 104 and 198 abnormal cells from datasets $\mathscr {A}$ and $\mathscr {B}$. These anomalies are manually annotated thanks to the original videos from which the dry mass time series were extracted. All cells detected in dataset $\mathscr {A}$ are labeled. On dataset $\mathscr {B}$, 104 cells are randomly drawn from the 198 detected as abnormal by the model. Only those cells are annotated to better compare the results on both datasets. We identified three main causes for the model to raise an anomaly:

(i) The cell, flagged as abnormal by StArDusTS has an abnormal behavior. Such behavior are discussed in detail after and are referred to as cellular anomalies or biological anomalies.
(ii) The acquisition system, including the lensfree microscope, the reconstruction algorithm, the segmentation algorithm and the tracking one, resulted in an error on the input fed into StArDusTS. Such anomalies are referred to as acquisition anomalies.
(iii) The cell seems to have a normal behavior, from the video and available time series perspectives. Such anomalies are False Positives (FP) in the detection.

Two more classes emerged from the manual annotation of anomalies. On the one hand, some abnormal cell behaviors mislead the acquisition system, therefore leading to erroneous times series. Such detected anomalies are both acquisition AND biologic anomalies. On the other hand, some anomalies are impossible for us to classify. As it impossible for us to say whether they are acquisition anomaly or biologic ones, they are called acquisition OR biologic anomalies.

Table 2 gives the details of the manual annotation of this experiment on both datasets $\mathscr {A}$ and $\mathscr {B}$. The precision is the ratio of the number of good detections to the total number of detections. StArDusTS algorithm was able to detect anomalies with a precision up to 96.2% on cells from Grenoble CEA and 83.5% on cells from Curie’s Institute.

**Table 2:** Distribution of annotated anomalies for experiment 1 on datasets $\mathscr {A}$ and $\mathscr {B}$. Underlined anomalies are biologic ones which are detailed in Table 3.

	$\mathscr {A}$	$\mathscr {B}$
Acquisition	49% (51)	31.1% (32)
Biologic	36.5% (38)	38.8% (40)
Acq and Bio	2.9% (3)	3.9% (4)
Acq or Bio	7.7% (8)	9.7% (10)
False positives	3.8 % (4)	16.5% (17)
Precision	**96.2%**	**83.5%**

Significant values are in bold.

**Table 3:** Manual annotation of biologic anomalies for experiment 1 on datasets $\mathscr {A}$ and $\mathscr {B}$.

	$\mathscr {A}$ (38)	$\mathscr {B}$ (40)
(a) Abnormal division	8 % (3)	15% (6)
(b) Abnormal growth	50% (19)	40% (16)
(c) Merging cells	26% (10)	5% (2)
(d) Too big cells	5 % (2)	20% (8)
(e) Dead cells	8 % (3)	20% (8)
(f) Others	3 % (1)	0% (0)

Most biological anomalies are different one from another. However, we propose here to group them into 5 major classes. The latter identified during the manual labeling of abnormal cells:

- Cells with an abnormal division, including cells that do not divide and stagnate on a plateau of dry mass, cells that divide asymmetrically or cells that divide in more than two daughter cells,
- Cells with an abnormal growth, which may include growth that is too long, too short, non-linear or with unexplained dry mass loss,
- Cells that merge with each other,
- Cells that are too big,
- Dead cells
- All other cellular anomalies.

**Figure 3:**
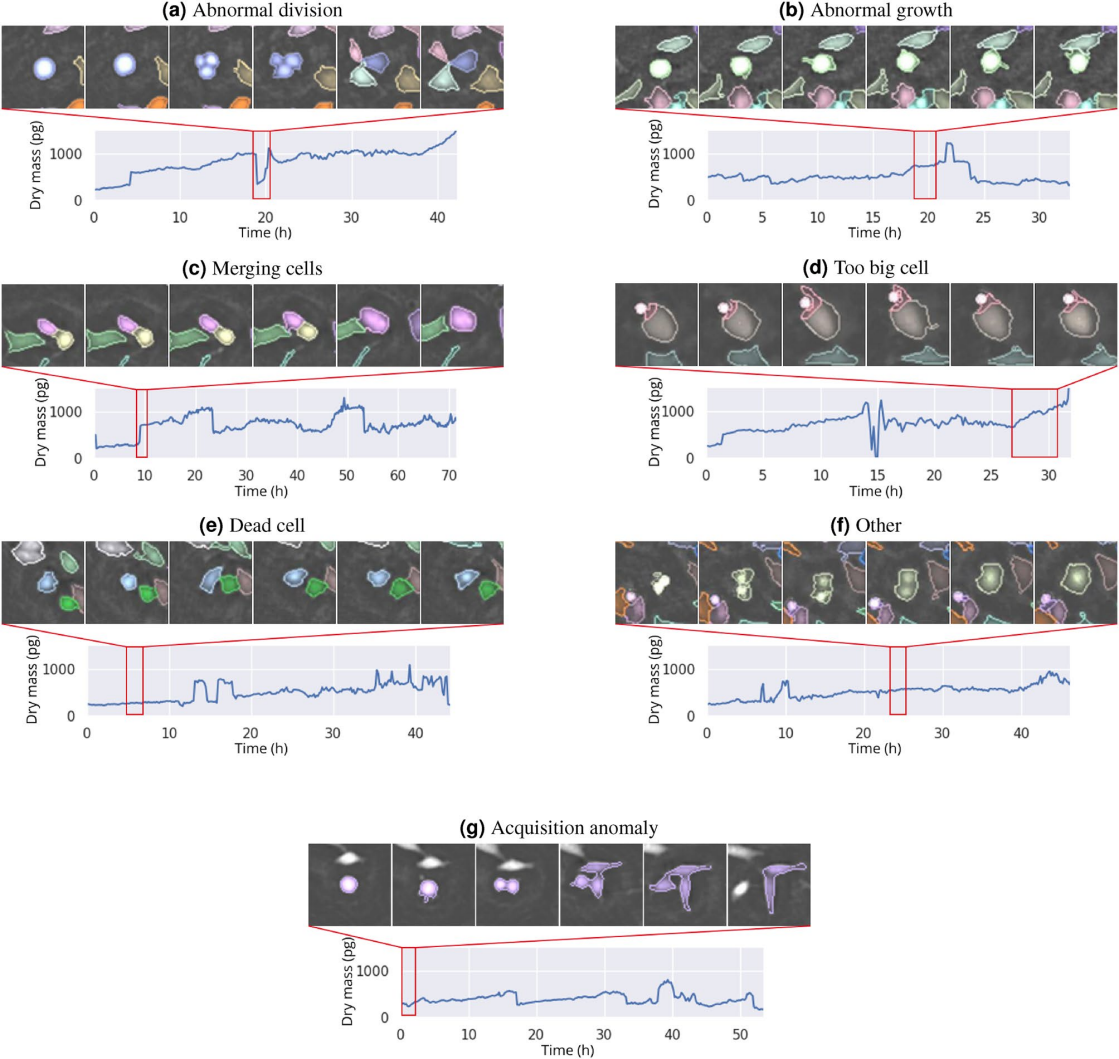
Time-lapse acquisition of adherent cells (Hela) that were detected as abnormal by StArDusTS. Each subplot depict an example of a subclass of anomalies. Every cropped image is $100\times 100 \mu$  m$^2$. The time between two images is 20 min. Cell tracking and cell segmentation are computed together to obtain time-lapse series. Each cell successfully tracked is depicted with a different color. The cell of interest is centered in the cropped image. Under each cell, the dry mass (in pg) time series from which the anomalies have been detected is printed. The time steps corresponding to the pictures are framed in red.

Table 3 shows the class distribution of these biological anomalies, both on cells from Grenoble CEA and Curie’s Institute. Figure 3a-f show the original acquisitions time-lapses (*i.e.* series of snapshots) of one example for each cellular anomaly class. For each time-lapse, we display the dry mass time series from which the anomalies were detected. Each instant corresponding to the images shown above are framed in red. Figure 3g focuses on an acquisition anomaly. More examples of all anomalies are available in the supplementary materials figure B. The videos from which these snapshots were extracted are also available as supplementary material.

### Experiment 2 : Generalization

We showed in the previous experiment that the StArDusTS model is able to detect anomalies in the dataset on which it has been train. To test its generalization capabilities, the model trained on $\mathscr {A}$ has been applied to a different set of HeLa cell acquisitions $\mathscr {B}$. HeLa cells were considered as a good model for detecting anomalies, as they often present one extra version of most chromosomes with up to five copies detected in a single cell^48^. Moreover, cells in *a* and in *b* were cultured and imaged under different protocols and laboratories, therefore increasing the differences between the phenotypes.

When conducting this experiment, the StArDusTS model identified 939 abnormal cells from the 7283 cells of dataset $\mathscr {B}$. Among those cells, 152 were already detected as abnormal is the previous experiment. This shows a strong consistency of the models in the detection of abnormal cells, since 76% of the cells detected in the first experiment were also detected as abnormal in this experiment. Tables 4 and 5 respectively show the distribution of the 104 manually annotated cells and the manual classification of biologic anomalies.

When the model was trained and tested on the same cells from dataset ($\mathscr {A}$), it detected 49% of acquisition anomalies and 36.5% of biological anomalies. During the generalization tests, StArDusTS detected 42.3% of acquisition anomalies and 43.3% of biological anomalies, proving the good generalization of the representation.

**Table 4:** Distribution of annotated anomalies for experiment 2.

Acquisition	42.3 % (44)
Biologic	43.3 % (45)
Acq and Bio	6.7 % (7)
Acq or Bio	3.8 % (4)
False positives	3.8 % (4)
Precision	**96.2%**

Significant values are in bold.

**Table 5:** Manual classification of the biologic anomalies of experiment 2.

(a) Abnormal division	20 % (9)
(b) Abnormal growth	49% (22)
(c) Merging cells	9% (4)
(d) Too big cells	7 % (3)
(e) Dead cells	11 % (5)
(f) Others	4 % (2)

StArDusTS model obtained a better performance on the anomaly detection for dataset $\mathscr {B}$ when trained on dataset $\mathscr {A}$ (precision of 96.2%) than when it was trained on dataset $\mathscr {B}$ itself (83.5% precision). A plausible explanation is the difference in image quality between the 2 datasets. This experiment also shows that the model trained on dataset $\mathscr {A}$ can be transferred to other datasets. Indeed, it kept the exact same precision of 96.2% regardless of whether it is tested on dataset $\mathscr {A}$ or $\mathscr {B}$.

Two conclusions can be drawn from this experiment : First, we showed that StArDusTS model is general enough to be able to detect anomalies in dataset it has not been trained on. Second, the performances of the model, and especially its precision, is highly impacted by the quality of the dataset used for training.

### Experiment 3: Controlled experiment

The previous experiments have shown the capability of StArdusTS for anomaly detection. However, dataset $\mathscr {A}$ and $\mathscr {B}$ could not be used for the recall evaluation. At this purpose, wild type and genetically modified fibroblasts were analysed to quantify the number of undetected anomalies and completely characterize the StArdusTS model.

To address this problem, we propose to artificially create a *“labeled”* dataset. The model is trained on dataset $\mathscr {C}$ and tested on both datasets $\mathscr {C}$ and $\mathscr {D}$. Cells from $\mathscr {D}$ are expected to be detected as abnormal ones since they are genetically modified. Thus, we can label cells from $\mathscr {C}$ as “normal” and cells from $\mathscr {D}$ as “abnormal” such that both precision and recall of the StArDusTS model can be measured.

This experiment allows the computation of the recall to 0.68 at the threshold $\tau _w$.

**Figure 4:**
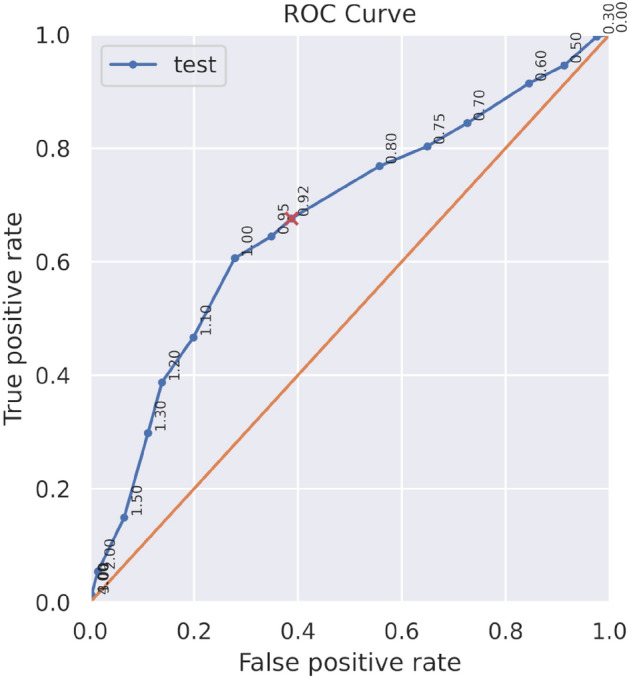
ROC curve of the anomaly detection on datasets $\mathscr {C}$ and $\mathscr {D}$. Each point is a threshold value of $\tau _w$ for the consideration of a window as abnormal. The red cross is the $\tau _w$ value computed from the training dataset $\mathscr {C}$ such that the time series outside the 95% interval of confidence are abnormal.

Figure 4 shows the ROC (Receiving curve for the detection of abnormal windows on datasets $\mathscr {C}_{\text {test}}$ and $\mathscr {D}$. The orange reference is the ROC curve of a random classifier. Each point is a threshold value of $\tau _w$. The red cross is the $\tau _w$ value computed from the training dataset $\mathscr {C}$ such that the time series outside the 95% interval of confidence are abnormal. It is however important to underline that the absolute values of precision and recall for the detections cannot be taken into account due to the strong hypothesis used for this experiment. Indeed, the assumption that all cells from dataset $\mathscr {C}$ are normal and $\mathscr {D}$ are abnormal might be biologically unrealistic.

The ROC curves show that choice of the $\tau _w$ value depending on the 95% confidence interval of the training dataset was made to have a good balance between the true and false positive rate. This is one of the best choice of threshold that could have been done since it is one of the points closest to the top left-hand corner of the graph.

## Conclusion

In this paper, we propose a model for Self-supervised Anomaly Detection on Time Series called StArDusTS, which we applied to the detection of abnormal cells from their dry mass over time series.

StArDusTS relies on the learning of a representation of normal cells with 1D convolutional neural network trained to predict the future cell dry mass. Thanks to self-supervised learning, the detection is processed without any human induced biases during training.

In a first experiment, we validate the anomaly detection abilities of the StArDusTS model by successfully detecting abnormal time series on two datasets with a precision up to 96.2%. We were able to manually identify 2 causes of anomalies, either being cellular anomalies or acquisition anomalies. Biological anomalies were then classified into 6 sub-classes. The acquisition anomalies that we report can be used to compare and improve acquisition pipelines if needed. A second experiment validated that the representation learned from one dataset is general enough to be able to detect anomalies from cells grown in another lab. Moreover, it shows that the results are even better than those obtained with a model trained on the same data. Finally, a third experiment with dummy labels of known anomalies was set up to validate the choice of the anomaly detector.

While the representation is learned only from the dry mass time series, the StArDusTS model could be extended for the prediction of multiple features such a cell area, thickness or speed. Adding more modalities such as cell area or thickness could bring more information about the cell population being analysed and thus allow the predictions of pathological changes.

### Supplementary Information

Supplementary Information.

## Data Availability

The data that support the findings of this study are available from the corresponding author upon request.
