# Supplementary material for: Detecting abnormal cell behaviors from dry mass time series
Source: Sci Rep. 2024 Mar 25;14:7053. doi: 10.1038/s41598-024-57684-w (PMC11350042; doi:10.1038/s41598-024-57684-w)
Supplement: Supplementary file 1 — Supplementary Information. [file 41598_2024_57684_MOESM1_ESM.pdf]

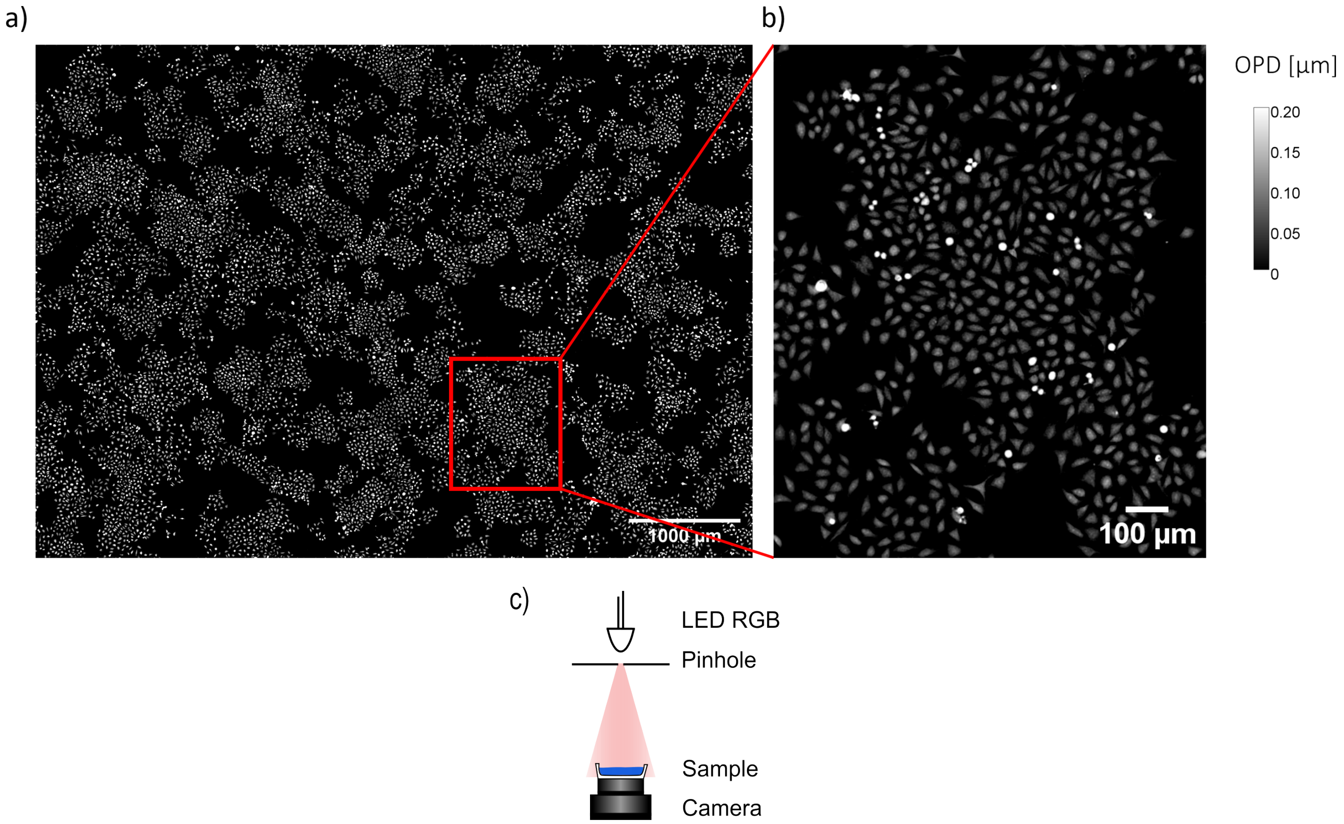

Figure A. Lensfree microscope acquisition of a HeLa cell culture. (a) Full field of view showing the final reconstruction of the optical path difference (OPD) image ($\sim 30\text{mm}^2$). (b) Detail of (a), red box. (c) A schematic of a typical lensfree microscope used to acquire such images

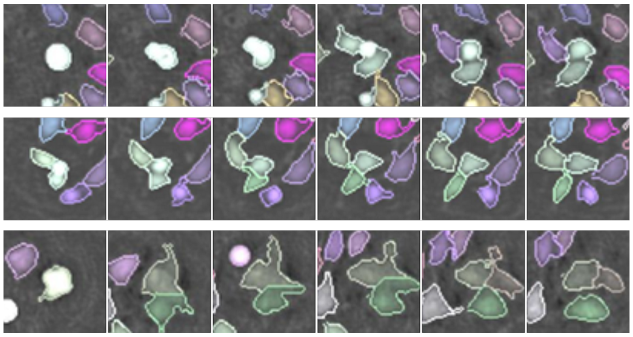

(a) Abnormal division

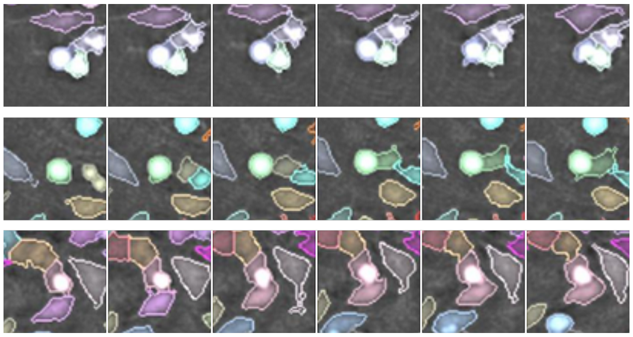

(b) Abnormal Growth

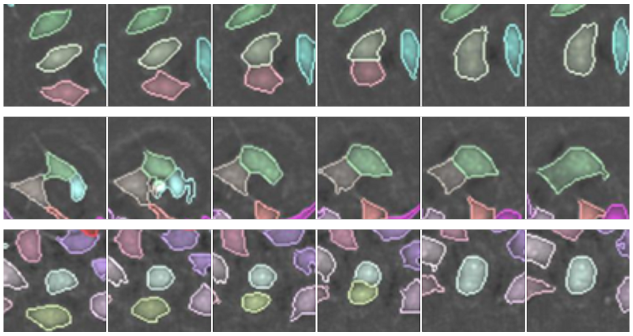

(c) Merging cells

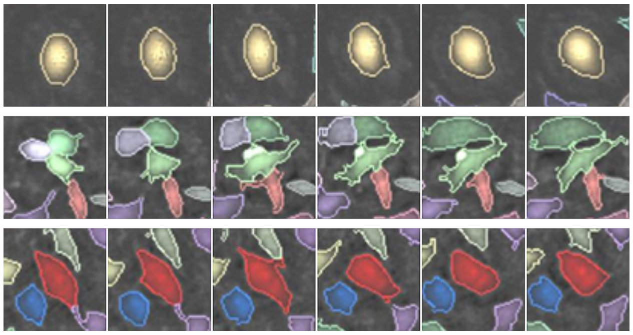

(d) Too big cell

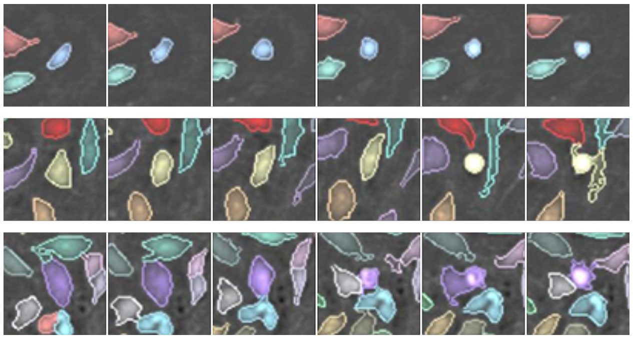

(e) Dead cell

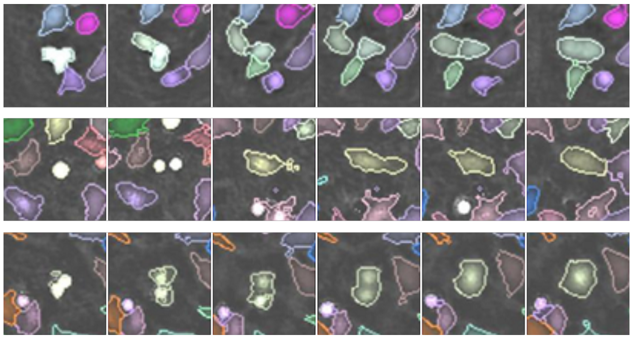

(f) Other

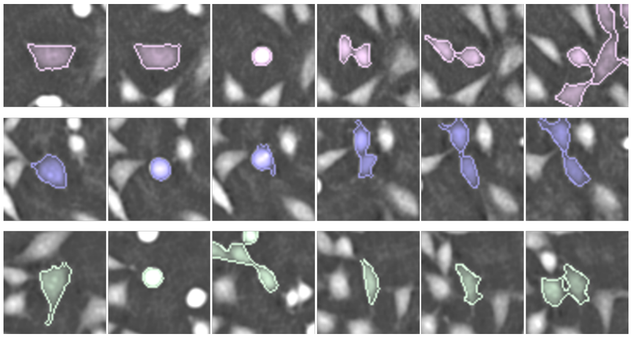

(g) Acquisition anomaly

Figure B. Time-lapse acquisition of adherent cells (Hela). Every cropped image is $100 \times 100 \mu m^2$. The time between two images is 20 minutes, except for Bg for which it is 40 minutes. Cell tracking and cell segmentation are computed together to obtain this time-lapse series. Each cell successfully tracked is depicted with a different color. The cell of interest are centered in the cropped image. The videos of this cells are available as supplementary material.

Legend for the videos provided as supplementary materials:

Videos figure3_a to figure3_f : The left column of graphs shows an example of 5 properties that can be extracted from the lensfree images, which are from top to bottom area, dry mass, motility, thickness and sphericity of the considered cell. Top left image is the phase reconstruction of an holographic image. The bottom left image is the segmentation of the cell of interest. On the top right image, the considered cell is centered in the image, each cell successfully tracked is depicted with a different color. The bottom left image is wide FOW (~ 1 mm^2) of the considered cell and its surroundings.

Their tracks are considered as biologically abnormal: (3a) Cell division leading to three cells instead of two. (3b) Cell mitosis exceeding 2 hours, which is twice the usual duration. (3c) Fusion of cells. . (3d) Very large cell exceeding 700 pg dry mass. (3e) Cell death. (3f) Other, here, cell fusion of the two daughter cells
